# Surgical Management of Chiari Malformation Type I in the Pediatric Population: A Single-Center Experience

**DOI:** 10.3390/jcm13123430

**Published:** 2024-06-12

**Authors:** Maria Sole Venanzi, Marco Pavanello, Mattia Pacetti, Francesca Secci, Andrea Rossi, Alessandro Consales, Gianluca Piatelli

**Affiliations:** 1 Neurosurgery Unit, IRCCS San Raffaele Scientific Institute, 20132 Milan, Italy; 2 Neurosurgery Unit, IRCCS Istituto Giannina Gaslini, 16147 Genoa, Italyconsales.alessandro@gaslini.it (A.C.); gianlucapiatelli@gaslini.org (G.P.); 3 Neuroradiology Unit, IRCCS Istituto Giannina Gaslini, 16147 Genoa, Italy; rossi.andrea@gaslini.it; 4 Department of Health Sciences (DISSAL), University of Genoa, 16126 Genoa, Italy

**Keywords:** Chiari malformation, syringomyelia, posterior fossa decompression, duraplasty

## Abstract

**Background**: Chiari malformation type 1 (CM-1) involves the cerebellar tonsils’ descent below the foramen magnum. In Chiari malformation type 1.5 (CM-1.5), both the cerebellar tonsils and the brainstem are herniated. Common symptoms include headaches and cervical pain, often associated with conditions like syringomyelia and hydrocephalus. Surgical treatment is not performed in asymptomatic patients, while the presence of syringomyelia represents an indication for surgery. **Methods**: This study retrospectively examined pediatric patients with CM-1 and CM-1.5 at Giannina Gaslini Hospital from 2006 to 2020, analyzing demographics, radiological findings, surgical interventions, and outcomes. **Results**: Out of 211 patients who underwent surgery, 83.9% were diagnosed with CM-1 and 16.1% with CM-1.5. Headaches were prevalent (69%) and cerebellar signs were noted in 29% of patients. Syringomyelia and hydrocephalus were present in 28.4% and 8% of cases, respectively. Intraoperative ultrasonography guided interventions, with 59.8% requiring bony and ligamentous decompression, and 27.1% undergoing duraplasty. **Conclusions**: The surgical treatment of CM-1/CM-1.5 involves posterior cranial fossa decompression. Choosing between bony decompression alone and its combination with duraplasty has always been controversial in the pediatric population. If we consider as surgical endpoint the restoration of cerebrospinal fluid (CSF) flux, intraoperative ultrasound may be a real-time helpful tool in orienting the surgical strategy, yet refinement with quantitative measures is needed.

## 1. Introduction

Chiari malformations (CMs) encompass a group of disorders distinguished by a downward displacement of cerebellar tonsils or vermis into the cervical spinal canal. The first comprehensive case report detailing this malformation was documented by Chiari in 1891 [1]. CMs can be distinguished into four types (1 to 4) [2]. Chiari malformation type 1 (CM-1) is characterized by abnormalities of the posterior cranial fossa (mostly small) and the caudal displacement of one or both cerebellar tonsils through the foramen magnum, using the diagnostic cut off of 5 mm from McRae line [3,4]. According to Ciaramitano P et al.’s recent classification, CM1-A presents syringomyelia at the full spine Magnetic Resonance Imaging (MRI), while in CM1-B there is no syringomyelia on MRI [4]. Chiari malformation type 1.5 (CM-1.5) represents a more severe variant of CM-1, characterized by both cerebellar tonsils and the brainstem being herniated below the foramen magnum [2,4].

The variety of symptoms of CM are due to the obstruction of cerebrospinal fluid (CSF) circulation because of anatomical abnormalities and to the compression of the neural structures or the presence of syringomyelia. The most common symptoms are headache [5,6] and cervical pain [7]. Headaches specifically attributed to CM1 are due to the disruption of the normal CSF flow at the foramen magnum. They are typically of short duration, occur at the back of the head, and are provoked by Valsalva maneuvers. Other headache types like migraines and tension headaches may coexist in children with CM1 and they are not expected to improve after surgery [7].

Compression exerted on the cerebellum and brainstem can result in a range of neurological symptoms: ataxia (20–40%), nystagmus (23–70%), dysfunction of lower cranial nerves (15–26%), sensory losses (30–92%), motor deficits, and signs of upper motor neuron lesions. In 20–30% of patients affected by CM-1, progressive scoliosis is present [8].

The most common finding in pediatric CM-1 or CM-1.5 is syringomyelia, documented in 67–80% of patients [9,10]. A dilatated central canal is frequently observed in the cervical and/or thoracic regions and it represents an indication for surgery. Additionally, other anomalies such as hydrocephalus, present in 10% of CM-1, or craniosynostosis or tethered spinal cord may be present [11]. These conditions may be addressed firstly by surgical treatment because it may resolve CM-1-related symptoms avoiding the need for decompression. 

## 2. Background of the Study

The surgical approach commonly employed for both CM1 and CM-1.5 is posterior fossa decompression. It can be performed alone or with associated duraplasty and/or tonsil resection [4,12]. A comprehensive analysis of the existing literature shows that the more aggressive the surgery, the longer and more prone to complications the operation. However, there seems to be a higher clinical improvement, with no need of a second intervention [4,12]. That is the reason why in adults, if surgery is indicated, decompression is always performed with duraplasty [13]. Analyzing surgical technical details, decompression should be wide enough on the foramen magnum, with the involvement of C1, but not too extended, thereby avoiding C2’s laminectomy to prevent craniovertebral junction instability [4].

Radiological evaluation, clinical history, and precise neurological examination are essential for patient selection for surgical intervention [14]. The recognized diagnostic cut-off of the caudal displacement of cerebellar tonsils through the foramen magnum is 5 mm from the McRae line but up to one third of patients with a radiological diagnosis of CM-1 may be asymptomatic [15]. Therefore, in the context of patient evaluation for surgery, greater emphasis is placed on the presence of symptomatic manifestations and the compression of neuronal structures at the level of the foramen magnum [14]. In asymptomatic patients, surgical treatment is not indicated, and since the natural history of CM-1 is still not clear, the treatment is limited to radiological and clinical follow-up. The presence of syringomyelia has been proven to be a criterion for surgical decompression while there is no shared consensus on operating symptomatic individuals without syringomyelia [4].

In contrast to the adult population, where bony decompression is always recommended with duraplasty, bone opening alone can be indicated in children [4]. Reflecting on our own experiences, we examined the intraoperative use of ultrasonography and systematically analyzed various aspects encompassing patients’ clinical profiles, surgical methodologies, and subsequent outcomes. The aim of this article is to illustrate how we employ this methodology in our decision-making process regarding surgical strategy for posterior fossa decompression.

## 3. Diagnostic Work-Up, Surgical Procedure, and Postoperative Course

According to the criteria reported above (see Introduction), CM-1 and CM-1.5 were sorted by means of a double-blind reading, including one neuroradiologist and one neurosurgeon. All patients with evidence of CM underwent a full spine MRI examination, with phase-contrast cine MRI to study the CSF flow. Neurophysiological studies were not routinely incorporated into our diagnostic and surgical management protocols.

All patients were positioned prone with bolsters under the shoulders and hips. Bony and ligamentous decompression with suboccipital craniectomy and C1 laminectomy alone or with the combination of dural delamination or with duraplasty with autologous graft were the possible surgical options. Tonsil coagulation was not standardly performed.

**Figure 1:**
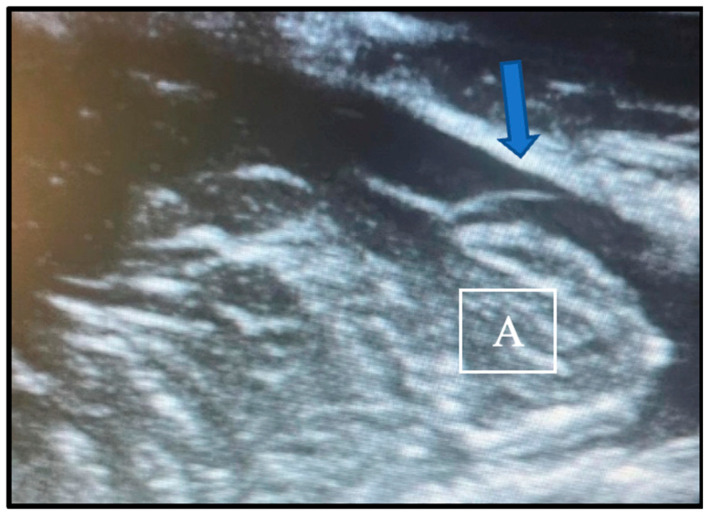
View of intraoperative ultrasound images depicting the absence of CSF flow (arrow) behind the cerebellar tonsils (A), indicative of the inadequate decompression of the structures.

We tailored the procedure of choice to the individual patient. In the presence of spinal syringomyelia, we always performed a dural and osteo-ligamentous decompression. In all other situations, however, we first performed a bony and ligamentous decompression and use intraoperative ultrasonography to evaluate the CSF dynamic and the pulsation pattern of the cerebellar tonsils. If the flow of cerebrospinal fluid in the foramen magnum region was recovered after the bony decompression, the duraplasty would not be carried out. The adequacy of decompression was confirmed by the presence of a CSF layer behind the tonsils and an antero-posterior pattern of tonsillar pulsation. Conversely, the need for dural opening was indicated by the absence of CSF flux and a “piston-like” movement pattern of the tonsils, characterized by vertical pulsations (see Figure 1). We relied solely on qualitative variables, not quantitative measures, with validation through cross-verification by the two operating neurosurgeons.

After dural closure, if deemed necessary, multiple Valsalva maneuvers were performed to check the continence of dura mater.

Intraoperatively, we used the SonoSite transducer, that emits soundwaves within a frequency range ranging from 1 to 5 MHz with a depth of up to 35 cm. To ensure optimal visualization, we positioned the transducer at an angle of approximately 60 degrees in the physiological saline acoustic window. This precise positioning allowed for the enhanced imaging and tracking of the CSF flow dynamics. We do not routinely use ultrasonographic imaging with pulse-wave Doppler.

Traditional postoperative pain control regimens relied on continuous opioid analgesia in conjunction with Ketorolac. Typically, these medications were delivered through patient- or nurse-controlled analgesia, spanning a duration of 3–4 days. Following this initial period, they were then switched to Paracetamol on demand.

A Computed Tomography (TC) scan was conducted for all patients within the immediate day following the surgical procedure. This CT scan served the dual purpose of evaluating the surgical outcomes and meticulously assessing for any potential complications that might have arisen.

Furthermore, at the three-month post-surgery mark, all patients underwent both a cerebral and a spinal MRI scan, as well as phase-contrast cine MRI. Our ongoing monitoring strategy involved scheduling regular MRI scans at intervals of every 12 months during the initial years post surgery, but they were repeated earlier than expected in the case of the onset of new symptoms.

## 4. Materials and Methods

We retrospectively examined all clinical charts and selected pediatric patients (aged 0–18 years old) affected by CM-1 and CM-1.5, who received medical care at the Giannina Gaslini Hospital in Genoa, Italy, during the period from 2006 to 2020. Patients exhibiting a concomitant diagnosis of achondroplasia or those with spina bifida, in the case of Chiari 2 (CM-2), were excluded from our study cohort.

Our retrospective examination involved a meticulous descriptive analysis encompassing various facets of the patients’ medical records: demographic details, radiological findings, and specifics regarding surgical interventions undertaken. Postoperative outcomes were evaluated using the Chicago Chiari Outcome Scale [16].

## 5. Results

**Figure 2:**
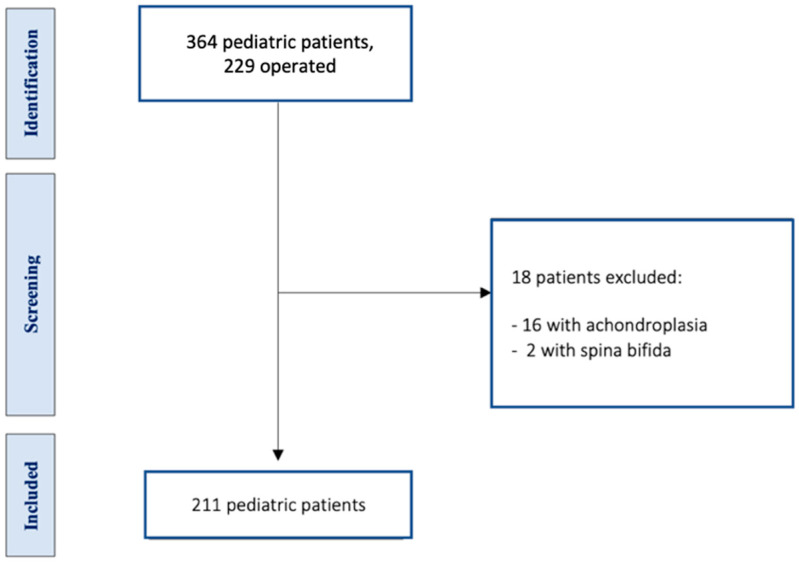
Flowchart with included and excluded patients.

Among 364 pediatric patients with caudal displacement of cerebellar tonsils through the foramen magnum (more than 5mm from McRae line) managed between 2006 and 2017 at the neurosurgery department of our pediatric center, we identified 229 who were surgically treated. Among them, 18 individuals were excluded, of whom 16 had achondroplasia in association with CM and 2 a diagnosis of CM-2 (with the presence of spina bifida), with a total of 211 patients for evaluation (see Figure 2).

The group included 122 males and 89 females, aged between 2 months and 18 years, with a mean age of 8.1 years. Children affected by CM-1 accounted for 83.9% and those affected by CM-1.5 for 16.1%.

Each patient included in the study underwent comprehensive brain and spine MRI scans. Specifically, out of the entire cohort, a subset of 37 patients, constituting approximately 16.2%, presented with symptoms indicative of sleep apneas. For this subgroup, additional diagnostic evaluation was conducted via polysomnography, to evaluate if these were of central origin or obstructive.

In total, 69% of the population exhibited headaches as a primary symptom, with 43% experiencing typical CM1 headaches and 26% reporting other types of headaches (see Table 1). Signs of cerebellar dysfunction, an unsteady gait, clumsiness in manual tasks, nystagmus, and ataxia were present in 29% of patients. Only 4.7% of individuals were asymptomatic at the initial visit in our center.

Syringomyelia was identified in 28.4% of the cases, exhibiting varying extent from affecting 2 vertebral bodies to encompassing the entirety of the spinal cord, with an average extension of 5.6 vertebral bodies. Specifically, only five patients presented syringomyelia that involved all the spinal cord. Additionally, hydrocephalus was associated in 8% of cases (see Table 1). In 6 of 17 patients with a diagnosis of hydrocephalus, the first treatment of this pathology was sufficient to relieve the symptoms associated with CM-1 or CM1.5.

Besides the anatomical anomalies of the posterior cranial fossa (such as platybasia) and anomalies of supratentorial structures, additional neuroaxial alterations were noted in 35 patients. Specifically, 8 cases involved non-syndromic craniostenosis, 4 of which underwent surgical treatment through skull remodeling; 11 cases exhibited craniovertebral junction malformations, 6 scoliosis, and 10 spinal dysraphism with tethered cord (see Table 1). Among the latter group, surgical detethering was deemed sufficient to relieve symptoms in only 2 cases. Additionally, two individuals were diagnosed with NF1 (Neurofibromatosis Type 1), and one had Ehlers–Danlos syndrome.

In our patient cohort, surgery was performed at a median of 21 months post initial evaluation (range: 5.1 months to 4.6 years).

Intraoperative ultrasonography evaluating CSF dynamics and cerebellar tonsil pulsation-guided treatment decisions. Bony and ligamentous decompression sufficed for 119 patients (59.8%), while 6 patients (3%) required a subsequent simple dural opening, and 74 (27.1%) underwent duraplasty with autologous grafts, with cerebellar tonsil coagulation in 3 of them.

One patient, previously operated elsewhere using osteo-ligamentous decompression, underwent expanded bony decompression with dural opening, experiencing post-surgery CSF leakage necessitating external lumbar drainage for a few days. Seven patients experienced postoperative complications. In three patients, surgery was performed in emergency: an evacuation of compressive subdural hematoma, a positioning of EVD (external ventricular drain) for acute hydrocephalus, and a removal of a broken subfascial drainage. Of the remaining four patients, two had a CSF fistula (in one patient, an external lumbar CSF drain was sufficient to solve the situation, while in the other, two surgeries to repair the leakage were mandatory), and two postoperative pseudomeningoceles, that disappeared at follow-up.

As expected, in dural and osteo-ligamentous decompression with or without autologous duraplasty, postoperative complications were significantly higher than in bony-ligamentous decompression (*p* < 0.05, see Table 2). Indeed, only one patient, in the case of the pure bony opening, had a rupture of subfascial drainage and needed reoperation. The data from the two populations are overlapping from a demographic perspective (refer to Table 3). In the first group, symptoms remained or worsened in 6.7% of cases, while in the second group, this occurred in 8.6%.

The median follow-up period was 4.2 years (range 2 months–10 years), and four patients were lost at follow-up. In only 7.1% of the 211 patients, symptoms remained stable or worsened. According to Chicago Chiari Outcome Scale, 9 patients were considered “impaired” (7 patients affected by CM-1 and 2 by CM-1.5), 6 were “incapacitated” (4 patients affected by CM-1 and 2 by CM-1.5), 101 had a “functional” outcome (76 patients affected by CM-1 and 25 by CM-1.5), and 95 had an “excellent” outcome (90 patients affected by CM-1 and 5 by CM-1.5). Among individuals with CM-1, 42.9% were classified under a “functional” outcome and 50.8% under an “excellent” outcome, while among individuals with CM-1.5, 73.5% were classified under a “functional” outcome and 14.7% under an “excellent” outcome.

In all patients with syringomyelia, the spinal cavity was reduced in dimension at the last spinal MRI of follow-up, except for four patients in which it remained stable and in one where it augmented without the need of surgical intervention.

Due to insufficient decompression at follow-up (shown by an inadequate CSF flow at the foramen magnum level and a persistence of symptoms), in three patients, a further operation with dural decompression and plasty was performed after the first bony and ligamentous opening, and in three others, an expansion of the previous osteo-dural decompression with the coagulation of tonsils was carried out.

## 6. Discussion

Since the symptoms and signs of CM are related to CSF obstruction and the compression of the cerebellum, brainstem, and spinal nerves, the standard surgical treatment consists of the decompression of posterior cranial fossa, releasing the cranial–cervical interface [17]. It is possible to conduct an osteo-dural decompression, with an opening of the dura mater and performing the duraplasty at the same time, in order to reduce surgical complications [18].

The degree of decompression in the pediatric population has always been a matter of controversy. Some neurosurgeons suggest that bony decompression alone is sufficient to restore the CSF flow [19], while others strongly believe in the superiority of osteo-dural decompression and duraplasty [20]. Regardless of the type of surgery used, the purpose is to restore the CSF dynamics and circulation through the foramen magnum.

Although a conspicuous number of theories have emerged to explain syringomyelia in Chiari, an ineffective CSF circulation seem to be at the basis of cervical syringomyelia [21]. And this is the reason why, in the presence of syringomyelia, an osteodural decompression with graft is always preferred. Indeed, in our series, in the majority of patients with syringomyelia who underwent surgery, the spinal cavity decreased in dimensions or completely disappeared at the MRI of follow-up. This concept is also underlined by a few publications where the authors showed how placing a stent from the fourth ventricle to the cervical subarachnoid space could promote the circulation of CSF and improve syringomyelia [22,23,24,25,26].

As in the adult population, the primary objective of surgery in children is to decompress the cerebellar tonsils, thereby mitigating the effects of tonsillar herniation through the foramen magnum and restoring normal CSF flow at the craniocervical junction [4]. If we consider this endpoint, i.e., the restoration of CSF flux, it would be rational to propose to all patients, whether affected by CM-1 or CM-1.5, an osteo-dural decompression as primary treatment. However, as also demonstrated by our case series, the dural opening is usually associated with a higher number of postoperative complications compared to bony decompression alone. Therefore, there is the need for a dynamic tool to measure CSF circulation to tailor decompression to the individual case.

Although MRI can depict CSF dynamics, as shown by Liu B et al. with electrocardiography-gated phase-contrast MRI [22], applying this technology in the operating room to choose the proper surgical strategy brings considerable difficulties. Intraoperative ultrasound is an efficient and inexpensive alternative to MRI, and its intraoperative use is much more feasible.

Several studies have utilized intraoperative ultrasonography to evaluate decompression success in patients with CM and associated conditions like syringomyelia [27,28,29]. Isu et al. observed that in six of seven patients affected by CM-1, the removal of the outer dural layer led to clinical improvement based on intraoperative ultrasound findings [28]. Navarro et al. employed ultrasonography in 72 pediatric patients to determine the extent of surgical decompression [28].

In our experience, the intraoperative decision not to undertake the opening of the dura mater and perform duraplasty was based on each surgeon’s subjective interpretation of the ultrasonography findings. The fact that this decision was based on non-quantitative and, therefore, non-systematic variables stands as a big limitation of our study. In order to ensure the replicability and reliability of intraoperative ultrasonography, there is a need to develop more studies with identified numerical variables.

Our analysis focused on the assessment of the CSF layer behind the tonsils, and their pulsation pattern represents a huge simplification of the pathophysiology of CM. According to the Monro–Kellie law, the “mobile compliance” of CSF within the intracranial space is somewhat constrained in CM, primarily attributed to a reduction in CSF circulation at the level of the foramen magnum. The vertical movement of the tonsils compensates for this local hydrodynamic disturbance in the interest of preserving intracranial cerebral pressure [30,31]. Indeed, clinical improvement seems to correlate with a decrease in the pulsatility of tonsils, as Capel et al. showed in their study [32].

Previous studies on animals, conducted with invasive methods, have shown that CSF flow is bidirectional: directly related to cardiac circle. In the systolic phase, since the cerebral flow increases with consequent cerebral expansion, CSF flows from the brain to the vertebral canal, while when the hearth muscle relaxes itself, the venous return is augmented and the cerebral blood reduced, causing the CSF to flow toward the cranial compartment. Breathing may also slightly influence CSF flow: during inhalation, thoracic pressure decreases, causing the blood to flow out and the CSF to flow in the brain, while on exhalation, the CSF in the cerebral ventricle flows in the caudal direction through the aqueduct. Since the thoracic pump influences blood and CSF dynamics, different respiration modes, such as the mechanical one under general anesthesia, noticeably affect CSF flow [33]. Furthermore, the impact of body posture on intracranial hydrodynamics is dramatic and has to be considered when the patient is operated in the lying-prone position, as attested by Alperin et al. in their MRI flow study [34,35].

One limitation of our retrospective study is the non-utilization of ultrasonographic imaging with pulse-wave Doppler. Indeed, cellular particles and proteins, that are contained inside CSF, can determine Doppler signals in ultrasonography. It has been possible since 1999 to complement ultrasonographic imaging with it to measure CSF velocity. Doppler ultrasonography faces technical challenges when imaging CSF flow. Unlike blood, CSF has distinct characteristics, including a minimal cell and protein content, which hinders ultrasound wave reflection. Additionally, its circulation involves a low-velocity, nonhomogeneous flow through irregular spaces. Overcoming these difficulties requires the use of high-performance equipment capable of maximizing Doppler sensitivity [36].

Cui LG et al. highlighted the role of Doppler ultrasonography in detecting CSF flow velocity and its variation during posterior fossa decompression. In their study, following single cranial bone decompression, CSF flow was absent (with a flow velocity of 0 cm/s) in 16 out of 20 patients. Weak frequency spectral signals of flow, ranging from 2.13 to 5.26 cm/s, were detected in three patients, while a distinct CSF flow (5–7 cm/s) was observed in one patient. In the latter patients, a pure bony decompression was effective without the need of further duroplasty. For the remaining patients, after additional duraplasty, an enhanced CSF flow with a clear frequency spectrum profile was observed, characterized by a bidirectional flow pattern and peak velocities ranging from 4 to 13 cm/s [17]. According to Milhorat et al., optimal CSF rates were achieved post decompression, with lower mean peak velocities ranging from 3 to 5 cm/s (compared to 0 to 0.8 cm/s before decompression). This improvement was also evidenced by the visible, unrestricted, and pulsatile flow of CSF from the fourth ventricle into the dorsal cervical theca [36].

## 7. Fine Modulo

In our institution, the percentage of operated patients with CM-1 or CM-1.5 is substantially higher compared to the figures documented in the literature: 63% versus 15% [17]. The reason lies in the fact that our institution is a secondary-level center. Patients initially consult their primary care physician or pediatrician before subsequently being referred to our center at an early stage. Upon arrival, an initial screening has been already conducted based on their presenting symptoms and the potential requirement for surgical intervention.

In our pediatric center, we offer surgical treatment to all symptomatic patients. The mean age of diagnosis is 8 years old, as in our patients’ series, although the range is wide and covers all pediatric ages [17].

Children with CM-1 or CM-1.5 may present a heterogeneous spectrum of symptoms and signs. The percentage of headaches reported by us appears to be lower compared to the existing literature [17]. This variance can be attributed to the inclusion of patients under the age of 5, where there was a greater challenge in recognizing this particular symptom.

In contrast to the existing literature, our reported prevalence of syringomyelia associated with CM-1 or CM-1.5 is notably lower, standing at 28.4% compared to the documented figure of 57% [37]. Similarly, the incidence of hydrocephalus in our patient cohort is also comparatively lower, accounting for only 8% of cases, a figure that contrasts with the 10% prevalence outlined by Koueik et al. [38]. These two pathologies can often coexist without a clear understanding of the cause-and-effect relationship. In the absence of other associated etiologies, neurosurgeons tend to treat the hydrocephalus first: in our series, six patients who were treated firstly for hydrocephalus did not need a subsequent decompressive surgery.

Most patients with CM-1/CM-1.5 do not have an underlying genetic condition. However, several other conditions have been associated with CM-1/CM 1.5, including NF1 (in two patients of our series), growth hormone deficiency, Klippel–Feil syndrome, multisuture or syndromic craniosynostosis (non-syndromic craniosynostosis in eight patients), Ehlers–Danlos syndrome (in one patient), and other hypermobility disorders [17,39].

Our assessment of surgical treatment success hinges upon the relief or disappearance of symptoms experienced by the patients, prioritizing clinical improvements to radiological improvement, since there are no well-defined criteria relating to the latter.

## 8. Limitations of the Study

Our manuscript is deficient in providing a quantitative evaluation of tonsillar herniation. Another significant limitation of our study is its exclusive reliance on qualitative variables, thereby overlooking the inclusion of quantitative measures in our analysis of intraoperative ultrasonography.

## 9. Conclusions

Our surgical series, in which a good clinical and radiological outcome was achieved in most of the patients, demonstrates how real-time ultrasonography may be a safe guiding tool during the surgical decompression of the posterior fossa.

The utilization of real-time ultrasonography within our clinical practice has indeed proven to be an invaluable asset, offering intraoperative guidance during surgical interventions. By providing immediate and dynamic visualization, this imaging modality aids surgeons in making informed decisions regarding the mode selection for posterior cranial fossa decompression and assists in the restoration of physiological CSF circulation. This real-time guidance might play a pivotal role in minimizing the necessity for re-operation, mitigating the risk of an inadequate decompression of posterior cranial fossa. However, there exists an imperative need to refine this method by incorporating quantitative variables, as some previous studies have shown. The potential integration of these quantitative measurements, eventually with the use of Doppler, holds promise in standardizing the application of ultrasonography within surgical procedures.

## Figures and Tables

**Table 1:** Symptoms and anomalies associated with CM-1 or CM-1.5.

Chiari Malformation	CM-1	CM-1.5
Headaches	117	129
Cerebellar symptoms	53	8
Sleep apneas	22	15
Syringomyelia	48	12
Hydrocephalus	12	5
Non-syndromic craniostenosis	6	8
Craniovertebral junction malformations	10	1
Scoliosis	6	0
Spinal dysraphism with tethered cord	9	1

**Table 2:** Postoperative complications for types of CM decompression (Fisher’s exact test).

Postoperative Complications	Bony and Ligamentous Decompression	Osteo-Dural Decompression ± Duraplasty	
**Y**	1	6	*p* = 0.0142
**N**	121	71	
**Tot**	122	77	

**Table 3:** Populations of patients who underwent dural-osteo-ligamentous decompression and osteo-ligamentous decompression.

	Bony and Ligamentous Decompression	Osteo-Dural Decompression ± Duraplasty
Mean Age	8.0 y (4 m–18 y)	8.2 y (1 y–19 y)
Ratio M/F	1.4	1.3
FU	2.0 y (3 m–4 y)	1.3 y (3 m–4 y)

## Data Availability

The data presented in this study are available on request from the corresponding author. The data are not publicly available due to privacy.

## References

[1] Koehler P.J. (1991). Chiari’s description of cerebellar ectopy (1891). With a summary of Cleland’s and Arnold’s contributions and some early observations on neural-tube defects. J. Neurosurg..

[2] Giallongo A., Pavone P., Tomarchio S.P., Filosco F., Falsaperla R., Testa G., Pavone V. (2021). Clinicoradiographic data and management of children with Chiari malformation type 1 and 1.5: An Italian case series. Acta Neurol. Belg..

[3] Balestrino A., Consales A., Pavanello M., Rossi A., Lanteri P., Cama A., Piatelli G. (2019). Management: Opinions from different centers-the Istituto Giannina Gaslini experience. Childs Nerv. Syst..

[4] Ciaramitaro P., Massimi L., Bertuccio A., Solari A., Farinotti M., Peretta P., Saletti V., Chiapparini L., Barbanera A., Garbossa D. (2022). Diagnosis and treatment of Chiari malformation and syringomyelia in adults: International consensus document. Neurol. Sci..

[5] Nohria V., Oakes J. (1990). Chiari I malformation: A review of 43 patients. Pediatr. Neurosurg..

[6] Steinbok P. (2004). Clinical features of Chiari I malformations. Child’s Nerv. Syst..

[7] Tubbs R.S., Beckman J., Naftel R.P., Chern J.J., Wellons J.C., Rozzelle C.J., Blount J.P., Oakes W.J. (2011). Institutional experience with 500 cases of surgically treated pediatric Chiari malformation Type I. J. Neurosurg. Pediatr..

[8] Garzon-Muvdi T., Jallo G.I., Poretti A., Ashmawy R., Huisman T.A.G.M., Raybaud C. (2016). Chiari Type 1 Deformity in Children: Pathogenetic, Clinical, Neuroimaging, and Management Aspects. Neuropediatrics.

[9] Milhorat T.H., Chou M.W., Trinidad E.M., Kula R.W., Mandell M., Wolpert C., Speer M.C. (1999). Chiari I malformation redefined: Clinical and radiographic findings for 364 symptomatic patients. Neurosurgery.

[10] Ellenbogen R.G., Armonda R.A., Shaw D.W., Winn H.R. (2000). Toward a rational treatment of Chiari I malformation and syringomyelia. Neurosurg. Focus.

[11] George T.M., Higginbotham N.H. (2011). Defining the signs and symptoms of Chiari malformation type I with and without syringomyelia. Neurol. Res..

[12] Yang M., Niu H.-T., Jiang H.-S., Wang Y.-Z. (2022). Posterior fossa decompression and duraplasty with and without tonsillar resection for the treatment of adult Chiari malformation type I and syringomyelia. Medicine.

[13] Lin W., Duan G., Xie J., Shao J., Wang Z., Jiao B. (2018). Comparison of Results Between Posterior Fossa Decompression with and without Duraplasty for the Surgical Treatment of Chiari Malformation Type I: A Systematic Review and Meta-Analysis. World Neurosurg..

[14] Raybaud C., Jallo G.I. (2018). Chiari 1 deformity in children: Etiopathogenesis and radiologic diagnosis. Handb. Clin. Neurol..

[15] Aitken L.A., Lindan C.E., Sidney S., Gupta N., Barkovich A.J., Sorel M., Wu Y.W. (2009). Chiari type I malformation in a pediatric population. Pediatr. Neurol..

[16] Yarbrough C.K., Greenberg J.K., Smyth M.D., Leonard J.R., Park T.S., Limbrick D.D. (2014). External validation of the Chicago Chiari Outcome Scale. J. Neurosurg. Pediatr..

[17] Cui L.-G., Jiang L., Zhang H.-B., Liu B., Wang J.-R., Jia J.-W., Chen W. (2011). Monitoring of cerebrospinal fluid flow by intraoperative ultrasound in patients with Chiari I malformation. Clin. Neurol. Neurosurg..

[18] Shou J.X., Ma L., Song L.J. (2007). Surgical treatment of Chiari I malformation with syringomyelia. Neurosurg. Clin. N. Am..

[19] Li S.F., Zhou M.D., Jia D.Z. (2003). Pathogenic mechanism and surgical treatment of Chiari I malformation with syringomyelia. Chin. J. Nerv. Ment. Dis..

[20] Tubbs R.S., Webb D.B., Oakes W.J. (2004). Persistent syringomyelia following pediatric Chiari I decompression: Radiological and surgical findings. J. Neurosurg..

[21] Sekula R.F., Arnone G.D., Crocker C., Aziz K.M., Alperin N. (2011). The pathogenesis of Chiari I malformation and syringomyelia. Neurol. Res..

[22] Sun P., Zhou M., Liu Y., Du J., Zeng G. (2023). Fourth ventricle stent placement for treatment of type I Chiari malformation in children. Childs Nerv. Syst..

[23] Han R.K., Medina M.P., Giantini-Larsen A.M., Chae J.K., Cruz A., Garton A.L.A., Greenfield J.P. (2023). Fourth ventricular subarachnoid stent for Chiari malformation type I–associated persistent syringomyelia. Neurosurg. Focus.

[24] Szuflita N.S., Phan T.N., Boulter J.H., Keating R.F., Myseros J.S. (2021). Nonoperative management of enlarging syringomyelia in clinically stable patients after decompression of Chiari malformation type I. J. Neurosurg. Pediatr..

[25] Spennato P., Vitulli F., Tafuto R., Imperato A., Mirone G., Cinalli G. (2023). Fourth ventricle to spinal subarachnoid space stenting in pediatric patients with refractory syringomyelia: Case series and systematic review. Neurosurg. Rev..

[26] Liu B., Wang Z.-Y., Xie J.-C., Han H.-B., Pei X.-L. (2007). Cerebrospinal fluid dynamics in Chiari malformation associated with syringomyelia. Chin. Med J..

[27] Isu T., Sasaki H., Takamura H., Kobayashi N. (1993). Foramen magnum decompression with removal of the outer layer of the dura as treatment for syringomeylia occurring with Chiari I malformation. Neurosurgery.

[28] Navarro R., Olavarria G., Seshadri R., Gonzales-Portillo G., McLone D.G., Tomita T. (2004). Surgical results of posterior fossa decom- pression for patients with Chiari I malformation. Childs Nerv. Syst..

[29] Hida K., Iwasaki Y., Koyanagi I., Sawamura Y., Abe H. (1995). Surgical indication and results of foramen magnum decompression versus syringosubarachnoid shunting for syringomyelia associated with Chiari I malformation. Neurosurgery.

[30] Radmanesh A., Greenberg J.K., Chatterjee A., Smyth M.D., Limbrick D.D., Sharma A. (2015). Tonsillar pulsatility before and after surgical decompression for children with Chiari malformation type 1: An application for true fast imaging with steady state precession. Neuroradiology.

[31] Balédent O., Henry-Feugeas M.C., Idy-Peretti I. (2001). Cerebrospinal Fluid Dynamics and Relation with Blood Flow: A Magnetic Resonance Study with Semiautomated Cerebrospinal Fluid Segmentation. Investig. Radiol..

[32] Capel C., Padovani P., Launois P.-H., Metanbou S., Balédent O., Peltier J. (2022). Insights on the Hydrodynamics of Chiari Malformation. J. Clin. Med..

[33] Alperin N., Lee S.H., Sivaramakrishnan A., Hushek S.G. (2005). Quantifying the effect of posture on intracranial physiology in humans by MRI flow studies. J. Magn. Reson. Imaging.

[34] Laganà M.M., Di Tella S., Ferrari F., Pelizzari L., Cazzoli M., Alperin N., Jin N., Zacà D., Baselli G., Baglio F. (2022). Blood and cerebrospinal fluid flow oscillations measured with real-time phase-contrast MRI: Breathing mode matters. Fluids Barriers CNS.

[35] Liu P., Fall S., Balédent O. (2022). Use of real-time phase-contrast MRI to quantify the effect of spontaneous breathing on the cerebral arteries. NeuroImage.

[36] Milhorat T.H., Bolognese P.A. (2003). Tailored operative technique for chiari type I malformation using intraoperative color doppler ultrasonography. Neurosurgery.

[37] Albert G.W. (2021). Chiari Malformation in Children. Pediatr. Clin. N. Am..

[38] Tubbs R.S., Griessenauer C.J., Oakes W.J., Albright A.L., Pollack I.F., Adelson P.D. (2015). Chiari malformations. Principles and Practice of Pediatric Neurosurgery.

[39] Koueik J., DeSanti R.L., Iskandar B.J. (2021). Posterior fossa decompression for children with Chiari I malformation and hydrocephalus. Child’s Nerv. Syst..

